# Development of oral osteomucosal tissue constructs *in vitro* and localization of fluorescently-labeled bisphosphonates to hard and soft tissue

**DOI:** 10.3892/ijmm.2014.1802

**Published:** 2014-06-11

**Authors:** SUSAN BAE, SHUTING SUN, TARA AGHALOO, JU-EUN OH, CHARLES E. McKENNA, MO K. KANG, KI-HYUK SHIN, SOTIRIOS TETRADIS, NO-HEE PARK, REUBEN H. KIM

**Affiliations:** 1 UCLA School of Dentistry, Los Angeles, CA 90095, USA; 2 Department of Chemistry, University of Southern California, Los Angeles, CA 90089, USA; 3 UCLA Jonsson Comprehensive Cancer Center, Los Angeles, CA 90095, USA; 4 David Geffen School of Medicine at UCLA, Los Angeles, CA 90095, USA; 5 Kyung Hee University, Dongdaemungu, Seoul 130-701, Republic of Korea

**Keywords:** osteomucosal tissue constructs, soft tissue, hard tissue, bisphosphonates, osteonecrosis of the jaw, ulceration, toxicity

## Abstract

Bisphosphonates (BPs) are anti-resorptive agents commonly used to treat bone-related diseases; however, soft tissue-related side-effects are frequently reported in some BP users, such as oral or gastrointestinal (GI) ulcerations. BPs are stable analogs of pyrophosphate and have high affinity to hydroxyapatite, allowing them to bind to the bone surfaces and exert suppressive effects on osteoclast functions. However, the underlying mechanisms as to how bone-seeking BPs also exert cytotoxic effects on soft tissue remain unknown. In the present study, we investigated the localization of nitrogen-containing BPs (N-BPs) in hard and soft tissue using fluorescently-labeled N-BPs *in vitro*. We developed osteomucosal tissue constructs *in vitro* to recapitulate the hard and soft tissue of the oral cavity. A histological examination of the osteomucosal tissue constructs revealed a differentiated epithelium over the bone containing osteocytes and the periosteum, similar to that observed in the rat palatal tissues. Following treatment with the fluorescently-labeled bisphosphonate, AF647-ZOL, the osteomucosal constructs exhibited fluorescent signals, not only in the bone, but also in the epithelium. No fluorescent signals were observed from the control- or ZOL-treated constructs, as expected. Collectively, the data from the present study suggest that N-BPs localize to epithelial tissue and that such a localization and subsequent toxicity of N-BPs may be associated, at least in part, with soft tissue-related side-effects.

## Introduction

Bisphosphonates (BPs) are the most widely used anti-resorptive agents for osteoporosis, a skeletal disorder characterized by compromised bone strength with an increased risk of fracture (1,2). Some modern generations of BPs that contain a nitrogen group (N-BPs) are also important therapeutic agents for cancer patients. Intravenously administered, N-BPs improve the management and prevention of complications associated with bone cancer metastasis, such as pathological fractures, hypercalcemia, nerve root compression and spinal cord compression (3). N-BPs also prevent the bone loss caused by endocrine therapy in patients with breast and prostate cancer (4).

With frequent use of BPs in multiple clinical settings, a growing body of evidence supports the notion that some N-BPs cause side-effects that are specific to soft tissue (5). For example, one of the major side-effects of orally administered BPs is inflammation and ulcerations of the gastrointestinal (GI) tract and, to a lesser extent, oral mucosa. Indeed, GI toxicity is one of the major reasons patients drop out of N-BP clinical trials (6). Esophageal inflammation and ulcerations are frequently reported in N-BP users (7), and oral ulcerations are also observed in patients who suck BP tablets (8).

The long-term and high-dose users of certain N-BPs are also susceptible to developing osteonecrosis of the jaws (ONJ) following dental interventions (9). ONJ lesions are chronic wounds clinically manifested as exposed necrotic bone with unhealed overlaying oral mucosa for at least 8 weeks (10). The etiology of ONJ is hypothesized to be due to the inhibitory effects of N-BPs on osteoclasts, the bone resorbing cells important for bone remodeling and healing. However, due to the pleiotropic effects of BPs, several other hypotheses have been suggested. Among the multiple hypotheses explaining the etiology of ONJ is soft tissue toxicity in which BPs directly compromise the healing of the oral mucosal tissues (9,11). Clinical trials have indicated that approximately 80% of patients with ONJ have an invasive dental procedure, suggesting that trauma to hard and soft tissue is a precipitating etiological factor (12). In line with this finding, localized pressure to soft tissue underneath denture prostheses is a contributing risk factor for ONJ (13). Importantly, ONJ has also been observed on torus palatinus in a patient with long-term BP treatment even without trauma (14).

Although the aforementioned clinical observations suggest that N-BPs may have preferential cytotoxic effects to soft tissue, direct evidence of whether N-BPs localize to soft tissue, including the oral epithelium is lacking. Therefore, in the present study, we aimed to investigate the localization of N-BP in both hard and soft tissue. For this purpose, we developed *in vitro* osteomucosal tissue constructs that reconstitute the 3-dimensional (3D) histological architecture of soft and hard tissue and examined the localization of a fluorescently-labeled BP, a derivative of a parent BP that has been previously utilized and demonstrated in other BP localization studies (15,16).

## Materials and methods

### Cell culture and reagents

Human oral keratinocytes immortalized by HPV-16 whole genome (HOK-16B), normal human oral keratinocytes (NHOKs) and normal human oral fibroblasts (NHOFs) were cultured as previously described (11,17). The HOK-16B cells and NHOKs were grown in keratinocyte growth medium (KGM; Clonetech, San Diego, CA, USA), and the NHOFs were grown in Dulbecco’s modified Eagle’s medium (DMEM/199) (4:1) supplemented with 10% fetal bovine serum (FBS; Invitrogen, Carlsbad, CA, USA). Primary NHOFs which did not exceed 3 passages (p3) were used. The primary cells were obtained from a healthy donor following the approval of the UCLA Institutional Review Board (IRB). Zoledronate (ZOL) was obtained from LKT Laboratories, Inc. (St. Paul, MN, USA). Alexa Fluor 647, succinimidyl ester (AF647, SE) was purchased from Invitrogen.

### AF647-ZOL synthesis

AF647-ZOL was synthesized as previously described (18). Briefly, the approach is based on the attachment of a functionalized epoxide linker to the imidazolyl nitrogen of ZOL under mild reaction conditions (aqueous, near neutral pH and 21–40°C). The resulting drug-linker intermediate has an amino group which can be reacted with Alexa Fluor 647, succinimidyl ester in the next step. The final conjugate, AF647-ZOL, was purified by high-performance liquid chromatography (HPLC; purity >95%), and fully characterized by proton nuclear magnetic resonance (^1^H NMR) spectroscopy, phosphorus-31 nuclear magnetic resonance (^31^P NMR) spectroscopy, high resolution mass spectrometry (HRMS), ultraviolet-visible spectroscopy (UV-VIS) and fluorescence spectroscopy as previously described (18).

### Osteomucosal tissue constructs

The *in vitro* 3D oral osteomucosal tissue constructs were reconstituted as previously described with some modifications (n=2) (11). Briefly, calvariae from neonatal rats were placed on top of the acellular collagen matrix, onto which collagen matrix mixed with NHOFs was placed and allowed to solidify. After 3 days, the HOK-16B cells were plated on the top of the solidified collagen matrix and allowed to proliferate for an additional 4 days. The Transwell insert containing collagen matrices with NHOFs and HOK-16B cells was ‘air-lifted’ above the medium level, to allow the keratinocytes to undergo differentiation for 1 week. The osteomucosal tissue constructs were treated with ZOL- or AF647-ZOL-containing medium. ZOL-untreated samples were used as controls (CTL). Two weeks after airlifting, which was 1 week following treatment with ZOL or AF647-ZOL, the constructs were harvested.

### MTT assay

Cell viability in response to ZOL or AF647-ZOL treatment was accessed using MTT Cell Proliferation Assay (ATCC; Manassas, VA) according to the manufacturer’s instructions. Briefly, the NHOKs were plated into 96-well plates with 4×10^3^ cells in 200 μl medium/well. The following day, the cells were treated with increasing concentrations of ZOL or AF647-ZOL. Each condition was carried out in quadruplicate with 1 set of wells with no cells (control). On the 3rd day, 20 μl of 5 mg/ml MTT (yellow thiazolyl blue tetrazolium bromide) were added to each well. After incubating the cells for 3.5 h at 37°C, the medium was removed and 150 μl of MTT solvent was added. The plate was place on a shaker with a cover of tinfoil for 15 min. The plate was read in 570 nm using an ELx800 Absorbance Microplate Reader (BioTek, Winooski, VT, USA).

### Tissue processing and histological examination

The osteomucosal tissue constructs were photographed using a digital camera, and then separated into 2 sections. One half was processed as a frozen sample, and the other half as a formalin-fixed (10%), paraffin-embedded (FFPE) sample. The decalcification process was not performed to preserve the tissue-bound AF647-ZOL. The tissue embedding and sectioning for both the frozen and FFPE samples were processed in the UCLA Tissue Procurement Core Laboratory (TPCL). The samples were cut into 5-μm-thick sections. H&E staining was performed on the FFPE samples for histological analysis. The frozen samples were subjected to fluorescence detection.

### Detecting the fluorescently-labeled ZOL: AF647-ZOL

The frozen samples were cut into 5-μm-thick sections, and the sections (n=3 per group) were mounted with ProLong Gold Antifade Reagent (Invitrogen). The mounted slides were scanned at a 666-nm wavelength.

### Immunohistochemical (IHC) staining

IHC staining was performed as previously described (11). Briefly, the FFPE samples were sectioned at 4–5 μm and deparaffinized in a 60°C oven for 30 min followed by rehydration in xylene and an increasingly diluted series of ethanol. The rehydrated sections were unmasked in citrate buffer containing 6 mM citric acid and 34 mM sodium citrate at pH 6.0 above 95°C for 25 min. Endogenous peroxidase activity was blocked with 3% hydrogen peroxide for 15 min, and the slides were incubated with 10% normal goat serum in PBS for 30 min. The tissues were incubated with primary antibodies K14 (LL001; Santa Cruz Biotechnology, Inc., Santa Cruz, CA, USA) at 1:200 for overnight followed by respective secondary antibodies and HRP-Avidin. The stained tissues were developed using the 3,3′-diaminobenzidine (DAB) chromogen substrate (Vector Laboratories Inc., Burlingame, CA, USA) and mounted using mounting medium.

## Results

### Development of osteomucosal tissue constructs in vitro

**Figure 1:**
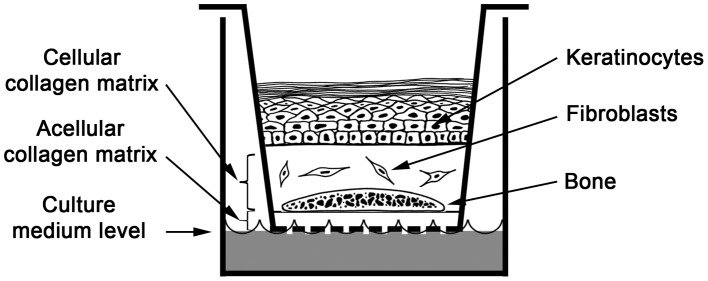
Schematic diagram of osteomucosal tissue constructs *in vitro*. Immortalized human oral keratinocytes (HOK-16B), normal human oral fibroblasts (NHOFs), and calvariae from rat pups were used to reconstruct the osteomucosal tissue constructs *in vitro* that mimic oral soft and hard tissue. A detailed procedure is explained in Materials and methods.

Although epithelial toxicity is suggested as a side-effect of BP use, direct evidence of BP localization to soft tissue is lacking. Thus, in this study, we aimed to investigate the localization of BPs in hard and soft tissue *in vitro*. We first constructed an *in vitro* model that mimics the 3D architecture of soft and hard tissue of the oral cavity in which the epithelial and connective tissue are situated immediately above the bone. To develop such osteomucosal tissue constructs, we modified the previously established raft culture systems by utilizing freshly prepared calvariae from rat pups (Fig. 1).

### Histological comparison between osteomucosal tissues in vitro and in vivo

**Figure 2:**
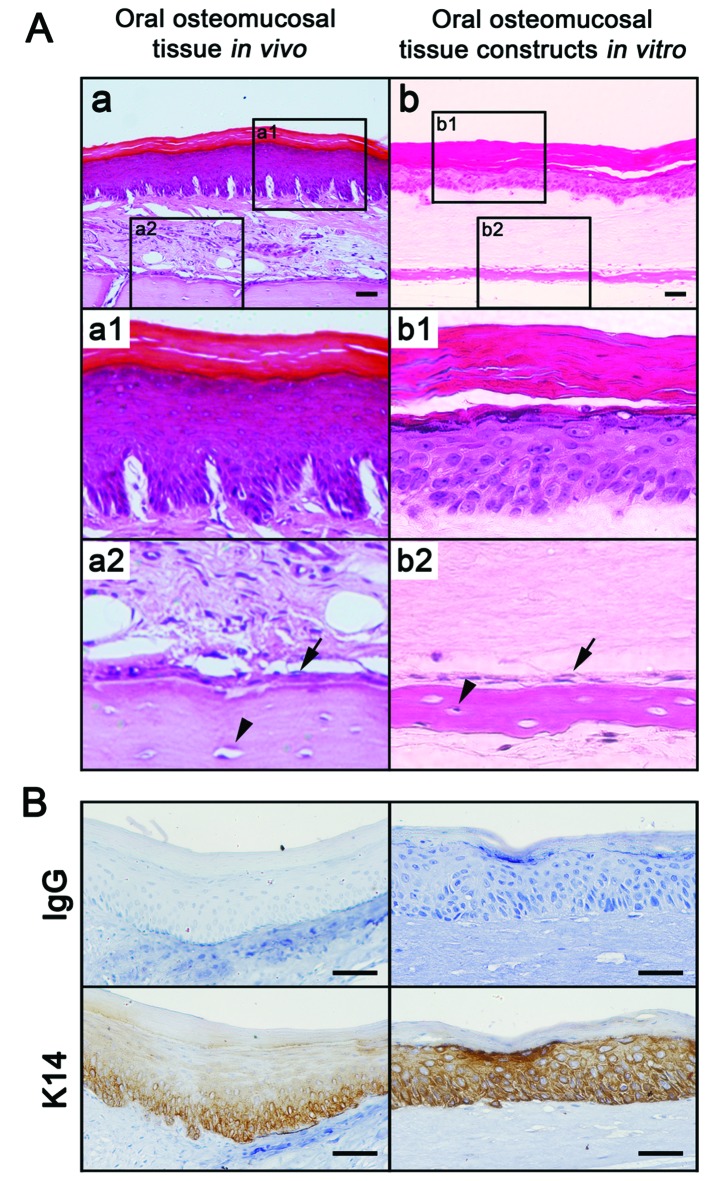
The osteomucosal constructs created i*n vitro* are histologically similar to oral osteomucosal tissue *in vivo*. (A) Osteomucosal tissues were obtained from the rat palates, and the histology was observed and compared with that of osteomucosal tissue constructs. The arrows indicate the periosteum and the arrowheads indicate osteocytes in the lacunae. The bar represents 100 μm. (B) Oral osteomucosal tissue from rats and the osteomucosal tissue constructs were stained with K14 antibody (1:200). The bar represents 100 μm.

Histological observation revealed that the *in vitro* osteomucosal tissue constructs have well-differentiated epithelial tissue that includes basal, spinous, granular and cornified layers (Fig. 2A-b and -b1). On the surface of the bone, the periosteum-like layer was evident (Fig. 2A-b2, arrow). In addition, osteocytes were observed in the lacunae (Fig. 2A-b2, arrowhead). Such histological findings were comparable to the *in vivo* oral osteomucosal tissue from the rat palates (Fig. 2A-a, -a1 and -a2), indicating that the *in vitro* osteomucosal tissue constructs recapitulated the *in vivo* histological architecture of the soft and hard tissue in the oral cavity.

Bone structures under the epithelium may prevent the proper delivery of nutrients supplied from the medium that is not in direct contact with airlifted soft tissue. Therefore, to further examine whether the differentiating epithelial layers are properly formed over the bone, we stained the osteomucosal tissue constructs with K14, a marker for basal keratinocytes in the epithelium. The epithelial tissues over the bone were positive for K14 staining, suggesting that the calvaria does not prevent the proper delivery of nutrients from the medium to the epithelial layer (Fig. 2B).

### AF647-ZOL localizes to hard tissue in the osteomucosal constructs

**Figure 3:**
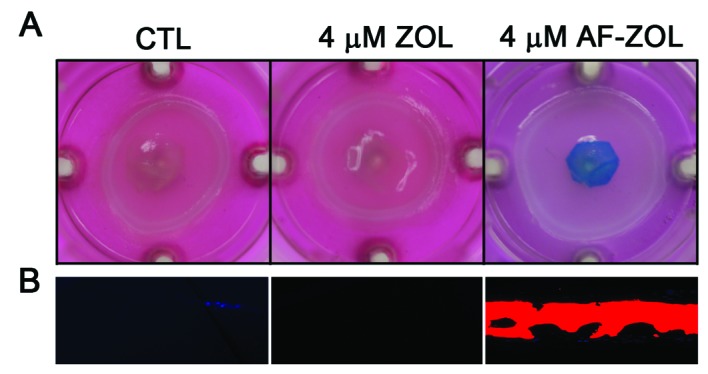
AF647-ZOL localized to the calvaria. (A) Osteomuocosal constructs were fed with medium containing no ZOL, 4 μM ZOL and 4 μM AF647-ZOL. The tissue constructs were collected after 2 weeks, and the frozen sections were prepared. (B) The fluorescent signals were observed at 666 nm using a fluorescence microscope (original magnification, ×200). DAPI staining was overlaid.

To examine whether AF647-ZOL localizes to the hard tissue, we prepared frozen undecalcified sections of the constructs and performed fluorescence microscopy. As expected, no fluorescent signals were observed in the control- or ZOL-treated constructs (Fig. 3, left and middle panels). However, intense fluorescent signals were observed from the bone in the AF647-ZOL-treated osteomucosal tissue constructs (Fig. 3, right panels), indicating that AF647-ZOL was primarily localized to the bone.

### AF647-ZOL also localizes to soft tissue

**Figure 4:**
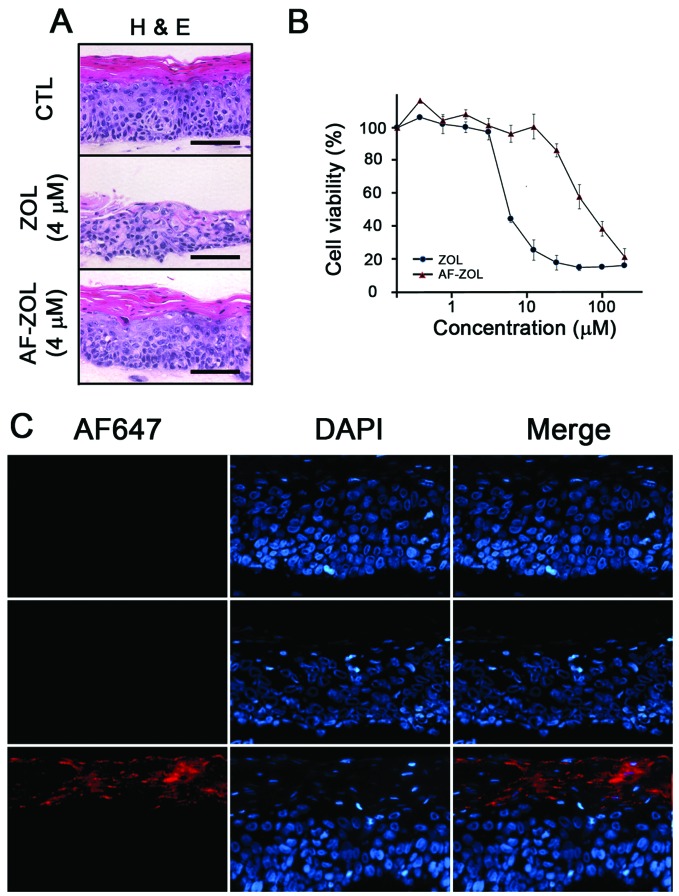
AF647-ZOL localized to the outer layers of the oral epithelium. (A) Osteomuocosal constructs were fed with medium containing no ZOL, 4 μM ZOL, and 4 μM AF647-ZOL, and the histology of the oral epithelium was observed by H&E staining of the FFPE samples. The bar represents 100 μm. (B) The NHOKs were subjected to MTT assay using various concentrations of ZOL and AF647-ZOL for 3 days. (C) The same constructs were prepared as frozen sections, and the fluorescent signals in the epithelial regions were observed at 666 nm.

To investigate whether AF647-ZOL localizes to areas other than hard tissue, we examined the localization of the fluorescent signals in the epithelium. When the histological analysis of the oral epithelial constructs was first carried out, all osteomucosal constructs exhibited differentiated architectures (Fig. 4A). Of note, the loss of cornified layers was evident in the ZOL-treated osteomucosal constructs. Previous studies have demonstrated a decrease in potency in fluorescent-labeled BPs (15,16). Therefore, we performed MTT assay to examine the viability of the NHOKs by ZOL and AF647-ZOL and found that ZOL was more potent in causing cell cytotoxicity (Fig. 4B), indicating that the difference in histological characteristics between the ZOL- and AF647-ZOL-treated osteomucosal constructs was due to the decreased potency in fluorescent-labeled ZOL. When we examined fluorescence in the epithelium, we found no fluorescent signals from the control- and ZOL-treated constructs (Fig. 4C, top and middle rows). However, fluorescent signals were observed in the epithelium of the AF647-ZOL-treated constructs (Fig. 4C, bottom row), indicating that the BPs also localize to soft tissue.

## Discussion

In the oral cavity, oral mucosal tissue is situated in close proximity to the underlying bone, without a prominent anatomical structure (e.g., muscle, fascia and fat) between these two entities. Therefore, an *in vitro* model that recapitulates this anatomical setting would be valuable in examining the association between the oral mucosal tissue and the underlying bone tissue in a controlled manner.

The oral mucosal tissue constructs created *in vitro* have been widely used to examine the forms and functions of the epithelium, including epithelial differentiation, wound closure and human papillomavirus (HPV) replication (19–21). Clinically, epithelial tissue constructs, or more widely known as cultured epithelial autografts (CEA), have been used to surgically restore large cutaneous defects, such as traumatic, burned and non-healing wounds (22,23), indicating that there are substantial functional and structural similarities between epithelial tissue constructs *in vitro* and actual epithelial tissue *in vivo*.

In the present study, we incorporated hard tissue components from rat calvariae and developed osteomucosal tissue constructs *in vitro* to mimic soft and hard tissue of the oral cavity. Using these osteomucosal constructs, we examined the localization of a fluorescently-labeled BP, AF647-ZOL. Consistent with previous reports (15,16), AF647-ZOL localized primarily to the bone surface. In addition, AF647-ZOL localized to the epithelium, suggesting that BPs may target not only the hard but also the soft tissue. The adverse effects of BPs on epithelial tissue, such as GI and oral ulcerations, have been frequently observed in clinical settings. Based on our findings, these epithelium-associated side-effects of BPs may be due to the localization of BPs to the epithelium.

BPs have ability to bind and chelate calcium (24). As such, BPs are known as bone-seeking agents due to the high calcium content in bone. Indeed, radiolabeled BP-derivatives, such as technetium 99m (99mTc) are clinically being used in nuclear medicine for bone imaging (25). Due to the calcium-seeking property of BPs, 99mTc is frequently observed in non-osseous but calcium-rich areas, such as chronic wounded or inflamed soft tissue (26). Similarly, the epithelium is highly enriched in calcium; the calcium gradients increase from the basal to the outer layers in which calcium contents are relatively high (27). Therefore, it is possible that the presence of fluorescently-labeled BPs to the epithelium, particularly in the outer layers is the result of the calcium-seeking characteristics of BPs.

At the molecular level, the primary mechanism of action of nitrogen-containing BPs (N-BPs) is to inhibit the mevalonate pathway by physically binding to and inhibiting the functions of farnesyl pyrophosphate synthase (FPPS), the key branch-point enzyme in this mevalonate pathway (28). Inhibition of FPPS by N-BPs is well-correlated with their anti-resorptive and apoptotic potency in osteoclasts (29,30). Some N-BPs, such as ZOL also target cancer cells to inhibit proliferation, and this inhibition is believed to be associated with FPPS (31–33). In epithelial biology, the mevalonate pathway is one of the key pathways in the maintenance of the physical barriers of the oral mucosa. *De novo* cholesterol biosynthesis and protein isoprenylation are indispensible processes for carrying out normal physiological functions, including the formation of stratified epithelium and differentiation into the stratum corneum to form a physical barrier (34). Indeed, several previous studies have demonstrated that N-BPs mediate adverse effects in oral epithelial cells via the mevalonate pathway (11,35). It is worthy to note that GI toxicity is more commonly observed with N-BPs (7,36), but is less commonly observed with non-N-BPs, such as clodronate or etidronate (37,38). Since N-BPs target the mevalonate pathway, it is conceivable that the ulcerative effects of N-BPs on epithelial tissue may utilize the same signaling cascade (35).

Several lines of evidence support the notion that oral mucosal defects may play a role in ONJ pathogenesis. First, N-BPs are known to inhibit the proliferation and migration of oral keratinocytes and fibroblasts *in vitro* (11,39,40). In addition, a recent study demonstrated that calcium potentiates the cytotoxic effects of N-BPs (41). Since the calcium concentration is high in the outer layers of the epithelium, it is possible that BP stored in the epithelium may be released while soft tissues undergo traumatic damages, affecting the nearby cells, including basal keratinocytes, impairing oral mucosal healing and leading to ONJ.

The present study has certain limitations to the utilization of *in vitro* osteomucosal tissue constructs. Although the culture medium was not in direct contact with the reconstructed oral mucosal tissues (Fig. 1), the continual replenishment of the medium with AF647-ZOL did not reflect the actual availability of ZOL in the body. In fact, circulating ZOL in the blood is rapidly cleared through renal secretion within hours of the first administration. In addition, the dose that we used may be significantly higher than what is used in the clinical setting. As such, BP localization to the epithelium warrants further investigation *in vivo*. Nonetheless, the present study using the newly established osteomucosal tissue constructs, which may serve as an important *in vitro* model in studying the association between soft and hard tissue in the oral cavity, and fluorescently-labeled BP suggests that BPs may localize to the outer-layer of the epithelium and cause soft-tissue toxicity.
